# West Nile Virus Infection in Plasma of Blood and Plasma Donors, United States

**DOI:** 10.3201/eid1510.080711

**Published:** 2009-10

**Authors:** Christina B. Planitzer, Jens Modrof, Mei-ying W. Yu, Thomas R. Kreil

**Affiliations:** Baxter Bioscience, Vienna, Austria (C.B. Planitzer, J. Modrof, T.R. Kreil); US Food and Drug Administration, Bethesda, Maryland, USA (M.-y.W. Yu).

**Keywords:** West Nile virus, intravenous immunoglobulins, IGIV, West Nile Virus, viral antibodies, neutralization tests, epidemiology, blood, blood products, viruses, dispatch

## Abstract

This study investigated the association of ongoing West Nile virus (WNV) infections with neutralizing antibody titers in US plasma-derived intravenous immune globulin released during 2003–2008. Titers correlated closely with the prevalence of past WNV infection in blood donors, with 2008 lots indicating a prevalence of 1%.

West Nile virus (WNV) is a flavivirus endemic to the United States; typically, hundreds of clinical cases of infection occur each year. The observed number of clinical WNV infections as collated by ArboNET (www.cdc.gov) and the incidence of asymptomatic WNV infections as shown by nucleic acid testing (NAT) of the US blood supply (*1*) indicate that ≈3 million WNV infections occurred in humans during 1999– 2008.

Because the immune system elicits WNV neutralizing antibodies in response to WNV infection, detectable levels of WNV neutralizing antibodies in the blood of persons with previous WNV infection is expected. Consequently, lots of immune globulin-intravenous (human) (IGIV) manufactured from plasma collected in the United States contain WNV neutralizing antibodies (*2*). Those IGIV lots, each prepared from several thousand plasma donations to ensure a broad spectrum of antibodies, can be used as an epidemiologic tool that enables the surveillance of thousands of persons in a community through analysis of comparatively few samples. In this study, we demonstrated the increasing trend of WNV-neutralizing antibody titers in lots of IGIV.

Comparing these titers with those of persons with confirmed past WNV infection provides an independent measure of the percentage of the US population previously infected with WNV. Several WNV vaccine trials are ongoing or imminent, so information about the prevalence of past WNV infection in the United States is valuable for planning the demonstration of vaccine efficacy. Low incidence and lack of highly WNV-endemic areas in the United States preclude classic vaccine field trials because of study size requirements and cost-logistics difficulties.

## The Study

The WNV neutralization titers of several US plasma–derived IGIV products (Gammagard Liquid/KIOVIG; Gammagard S/D/ Polygam S/D; Iveegam EN [Baxter Healthcare Corporation, Westlake Village, CA, USA]) and plasma samples obtained from US blood donors after a NAT-confirmed WNV infection were determined by an infectivity assay as earlier described (*2*), adapted to a classical microneutralization format (*3*). WNV neutralization titers (i.e., the reciprocal dilution of a 1:2 series resulting in 50% neutralization [NT_50_; detection limits <0.8 for undiluted IGIVs and <7.7 for 1:10 prediluted serum]) are reported as the mean ± SEM. An unpaired *t* test was used to evaluate whether titer differences between 2 groups were statistically significant.

Using an extrapolation derived from screening the US blood supply for WNV (*1*), we calculated the average annual number of WNV infections in the United States for 1999–2008. The total number of neuroinvasive cases reported for those years to the US Centers for Disease Control and Prevention (CDC) through ArboNET was multiplied by 256 (i.e., the factor between all WNV infections and neuroinvasive cases). The cumulative infection rate for each year during 1999–2008 was then calculated by dividing the infections occurring up to a specific year by the US population for that year (determined by US Census Bureau estimates [www.census.gov/popest/states/NST-ann-est.html]).

**Figure 1:**
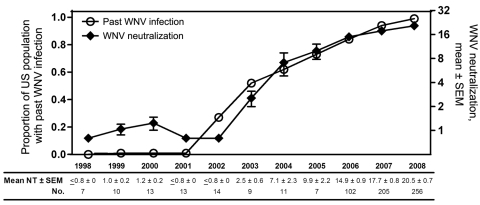
West Nile virus (WNV) neutralization titers of US plasma-derived immune globulin intravenous (human) (IGIV) lots by year of production and estimated percentage of the US population with past WNV infection by year. WNV neutralization titers were determined either for retention or lot release samples of 3 IGIV products produced during 1998–2005 or for a considerable proportion of Gammagard Liquid/KIOVIG lots produced during 2006–2008. Results are shown as mean ± SEM (limit of detection <0.8) by year of product release. For 5% of IGIV samples, titers were multiplied by 2 for comparison with the 10% IGIV samples at equivalent immunoglobulin concentrations. The percentage of the US donor population with past WNV infection was calculated from the number of neuroinvasive cases reported per year and the estimated ratio of neuroinvasive cases to total cases of WNV infection.

Although WNV was first introduced into the United States in 1999, only in 2003 did the mean WNV neutralization titers of IGIV lots released to the market start to increase markedly (Figure 1). According to extrapolations from the WNV screening of the US blood supply (*1*), by 2003, an estimated 0.5% of the US population had been infected with WNV, although most infections were asymptomatic.

A delay of ≈1 year occurs between the collection of plasma and the release of IGIV lots to the market; thus, the WNV-positive IGIV lots in 2003 reflect the larger number of WNV infections occurring in 2002. Using the same extrapolations from the US blood supply (*1*), we found that the ≈0.1% annual increments in the proportion of the US population with past WNV infection follow a straight line (r^2^ = 0.9996), generally paralleled by the mean WNV neutralization titers of IGIV lots. During 2005–2008, when large numbers of lots of a single IGIV product (Gammagard Liquid) could be analyzed, the WNV neutralization titer increased by 3.6 per year (r^2^ = 0.9793).

**Figure 2:**
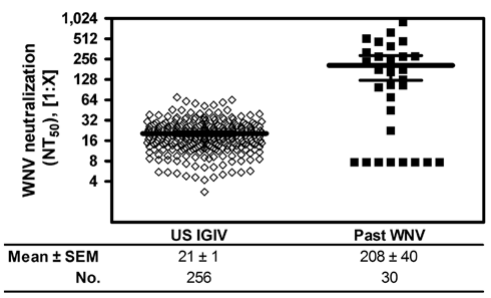
West Nile virus (WNV) neutralization by US plasma-derived immune globulin intravenous (human) (IGIV) released in 2008 and plasma from donors with past WNV infection (past WNV), confirmed by nucleic acid testing. WNV neutralization titers are shown as the mean ± SEM (limit of detection <0.8 for undiluted IGIVs and <7.7 for prediluted sera). NT_50_, 50% neutralization titer.

US plasma-derived IGIV lots released during 2008 showed variable WNV neutralization titers ranging from 2.8 to 69.8; mean ± SEM titer was 21 ± 1 (n = 256) (Figure 2). Compared with titers shown to be protective in an animal model of WNV infection (equivalent to >21 by the current assay) (*2*), ≈40% of the 2008 IGIV lots had higher titers.

Plasma obtained from persons with NAT-confirmed WNV infection had even higher titers; mean ± SEM titer was 208 ± 40 for 30 persons available for testing. When results were corrected for the immunoglobulin (Ig) G concentration in plasma (≈1%), compared with the 10% IGIV preparations, the mean neutralization titer of the plasma samples was ≈100× higher than that of the IGIV lots tested (2,080 vs. 21).

## Conclusions

The most comprehensive collation of information about the incidence of WNV infection in the United States is available from ArboNET. When that information is combined with information obtained from the nationwide screening of the blood supply for WNV RNA by NAT (*1**,**4**,**5*), the current prevalence of past WNV in the US population is estimated to be ≈1%.

Busch et al. has noted that large-scale, community-based serologic surveys are hardly feasible because of their expense and because WNV ELISA assays are possibly biased by cross-reactions with other flaviviruses (*1*). Nevertheless, 7 seroepidemiologic studies have been performed (*6**–**12*). Cumulatively, 5,503 persons were tested for WNV infection by ELISA, and the results have shown highly divergent seroprevalence rates ranging between 1.9% (*6*) and 14.0% (*10*).

The use of IGIV lots, each representing the serostatus of several thousand donors in 1 sample, makes seroepidemiology practical (*13*) because it allows a large donor population to be surveyed by analyzing comparably few samples. The use of a more complex yet functional virus neutralization assay minimizes concerns about cross-reactivity with flaviviruses of other serocomplexes (e.g., dengue virus) that occasionally circulate in the US population. Also, epidemiologic considerations render interference by St. Louis encephalitis virus, a flavivirus within the same serocomplex, highly unlikely (*2*). The specificity of the neutralization assay was confirmed by testing IGIV lots manufactured from European-derived plasma against tick-borne encephalitis virus, a flavivirus closely related to WNV and circulating in Europe. Although these lots contained high neutralization titers against tick-borne encephalitis virus, only 1 of 20 had a detectable neutralization titer of 5 against WNV (unpub. data).

In this study, we determined that the mean titer of samples obtained during 2003–2008 from persons with a confirmed diagnosis of WNV infection was 100× higher than the mean titers of IGIV lots produced in 2008. This determination provides an independent experimental measure of the frequency of past WNV infection in the general US population, as reflected by the plasma/blood donor community, and the results correlate well with results of previously published theoretical extrapolations (*1*), which estimated that ≈1% of the population has already been infected with WNV.

The increasing levels of WNV neutralizing antibodies in IGIV lots from US plasma and the particularly high titers in donors who have had a WNV infection suggest the possibility of preparing IGIV products with sufficiently high titers to be useful for WNV prophylaxis or treatment. Several ongoing or imminent WNV vaccine clinical trials stress the practical value of an independent confirmation of extrapolations that estimate the percentage of the US population with past WNV infection. Knowing the percentage of preexisting WNV seroprevalence as well as estimates of the mostly asymptomatic incidence rates (*14*) can be of vital importance in designing vaccine trials.
